# Nationwide investigation of eukaryotic pathogens in ticks from cattle and sheep in Kyrgyzstan using metabarcoding

**DOI:** 10.1371/journal.pone.0327953

**Published:** 2025-08-05

**Authors:** Singeun Oh, Nathalie Amvongo-Adjia, Hyun Jung Kim, Jun Ho Choi, Xavier Chavarria, Myung-hee Yi, Arwa Shatta, Bekbolsun Aknazarov, Ju Yeong Kim, Jung-Won Ju

**Affiliations:** 1 Department of Tropical Medicine, Institute of Tropical Medicine, Arthropods of Medical Importance Resource Bank, Yonsei University College of Medicine, Seoul, Republic of Korea; 2 Department of Tropical Medicine, Graduate School of Medical Science, Brain Korea 21 Project, Yonsei University College of Medicine, Seoul, South Korea; 3 Department of Global Health and Disease Control, Graduate School of Public Health, Yonsei University, Seoul, Republic of Korea; 4 Centre for Research on Health and Priority Diseases, Institute of Medical Research and Medicinal Plants Studies (IMPM), Yaounde, Cameroon; 5 Division of Vectors and Parasitic Diseases, Korea Disease Control and Prevention Agency (KDCA), Cheongju, Republic of Korea; 6 Faculty of Veterinary Medicine, Kyrgyz National Agrarian University Named after K. I. Skryabin, Bishkek, Kyrgyzstan; Van Yuzuncu Yil University Faculty of Veterinary Medicine: Yuzuncu Yil Universitesi Veteriner Fakultesi, TÜRKIYE

## Abstract

Ticks are significant vectors of bacterial, viral, and protozoan pathogens, impacting both public health and agriculture. In Kyrgyzstan, tick-borne diseases are a growing concern for livestock and human health. While bacterial and viral pathogens are widely studied, and limited previous investigations have focused on specific *Babesia* and *Theileria* species in certain host animals, comprehensive data on tick eukaryotic microbiota and potential pathogens across diverse hosts nationwide is scarce. To address this gap, our study provides the comprehensive nationwide assessment of the potential protozoan pathogens in ticks from cattle and sheep, analyzing data of *Babesia* and *Theileria* at the genus level. We collected 472 tick samples from cattle and sheep across seven regions of Kyrgyzstan (March-July 2022). Tick species were identified via microscope and Sanger sequencing (mitochondrial COI gene). Eukaryotic microbiota was analyzed using 18S rRNA V9 NGS. Sanger sequencing identified five genera and 11 tick species. NGS analysis revealed *Babesia* (13.3%) and *Theileria* (12.7%) as among the most prevalent protozoa detected at the genus level. *Babesia* was significantly more prevalent in nymph-stage ticks and those collected from sheep, whereas *Theileria* was detected across a broader range of tick species and host animals, showing less variation across life stages. No significant differences in prevalence were observed based on tick sex or the number of hosts in the tick life cycle. Regionally, *Babesia* detection was highest in the Osh region, particularly in ticks collected from both cattle and sheep. This is the first comprehensive nationwide analysis of tick eukaryotic metabarcoding study in Kyrgyzstan focusing on pathogenic protozoa detected at the genus level. Findings provide crucial baseline data on *Babesia* and *Theileria* geographic and host-specific prevalence. Understanding these information is essential for advancing future research and supporting the development of effective surveillance and control strategies against babesiosis and theileriosis in regional livestock.

## Introduction

Ticks are significant vectors of disease in humans and animals worldwide. Their ability to transmit various pathogens, including bacteria, viruses, and parasites, poses a serious threat to public health and agriculture [1–3]. In recent years, the emergence and resurgence of tick-borne diseases (TBDs) have increased, driven by factors such as climate change, globalization, and habitat disruption [4,5].

Although bacterial and viral tick pathogens have been extensively studied [6–8], the eukaryotic component of the tick microbiota remains relatively unexplored. Understanding the diversity and distribution of these eukaryotic microorganisms is crucial for comprehending their potential roles in pathogen transmission, tick biology, and host–parasite interactions.

*Babesia* and *Theileria* are protozoan parasites that pose serious health risks to both livestock and humans [9]. These pathogens cause diseases such as bovine babesiosis and theileriosis, leading to substantial economic losses in agriculture due to reduced productivity and higher veterinary costs [10]. Understanding their detection rates and distribution in tick populations is crucial for protecting public health and agricultural stability in regions where livestock practices facilitate pathogen transmission.

Kyrgyzstan, a Central Asian country with rich biodiversity and diverse ecosystems, possesses a unique geographical position that fosters optimal conditions for tick proliferation and disease transmission [11,12]. Recent advances in molecular techniques, such as next-generation sequencing (NGS), have enabled studies of the diversity of microorganisms and pathogens carried by ticks [8]. However, studies focusing on eukaryotic microorganisms using NGS remain limited, creating a knowledge gap in understanding their role in disease transmission in Kyrgyzstan.

In this study, we aimed to fill this gap by using NGS-based DNA metabarcoding to explore the eukaryotic microbiota of ticks from various regions of Kyrgyzstan, focusing on the V9 region of the 18S rRNA gene, a common marker in eukaryotic studies [13–15]. To investigate potential ecological drivers of pathogen prevalence, we examined the influence of host species, tick life stage, sex, and geographical region, as these factors have been shown to influence pathogen transmission dynamics [9–12,16,17].

To our knowledge, this is the first comprehensive nationwide study to use metabarcoding to analyze the relationships between tick species, developmental stages, regions, and host types. By combining high-throughput techniques with ecological data and diagnostic methods, this study offers valuable insights that could guide tick management strategies, public health initiatives, and diagnostic advancements, ultimately improving the control of TBDs in Kyrgyzstan.

## Materials and methods

### Sample collection and DNA extraction

**Fig 1:**
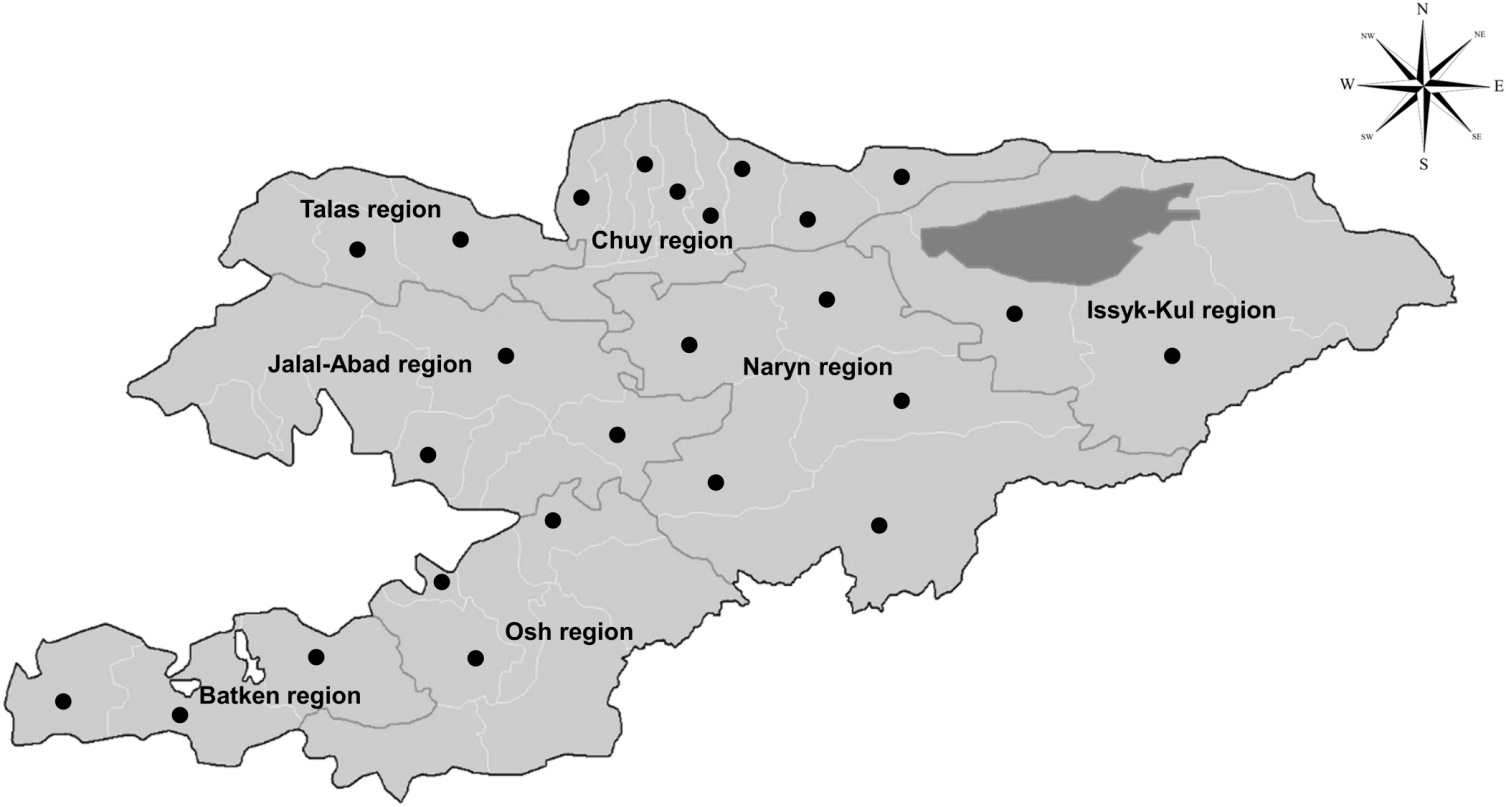
Locations of tick sample collection by district across Kyrgyzstan. A total of 472 tick samples were collected from seven regions. The dots indicate the districts where the samples were collected. The base map was adapted for illustrative purposes from administrative boundary data provided by the United Nations Office for the Coordination of Humanitarian Affairs (OCHA).

Ticks were collected from cattle and sheep grazing in pastures by careful removal with tweezers between March and July 2022. A total of 472 tick samples were collected from cattle and sheep across seven regions of the country (Fig 1). After collection, the ticks were submerged in 70% ethanol and stored at −20°C until processing. Their species, sex and life stages were determined under a dissecting microscope (Olympus, Tokyo, Japan) using a morphological classification key [18]. Tick life stages were categorized as nymph or adult, as these stages can differ in feeding habits and their ability to transmit pathogens. Larval ticks were not collected during this survey, as they are rarely found on large animal hosts and are often too small to be reliably identified in field studies [19]. The samples were then immediately frozen at −80°C until DNA extraction and molecular assays were performed.

Genomic material from ticks was prepared following the method described by Jung et al. (2024) [20]. Briefly, each tick was homogenized in a Reinforced Bead Tube (Zirconia 3 mm, Clear tube) using the MagMAX™ DNA Multi-Sample Ultra 2.0 Kit (Applied Biosystems, Waltham, MA, USA) and processed with the KingFisher Flex system (Thermo Fisher Scientific, Waltham, MA, USA) and a Precellys Evolution homogenizer (Bertin Technologies, Bretonneux, France). The homogenization was performed twice for 30 s at a speed of 4.5 m/s, followed by centrifugation for 10 min at 12,000 × g. Subsequently, the supernatant was transferred to a new sterile microtube, and DNA was extracted according to the manufacturer’s instructions. DNA concentrations were measured by spectrophotometry using an Implen Nanophotometer (Implen, Munich, Germany).

### Sanger sequencing and molecular identification of tick species

Molecular identification of tick species was conducted by amplifying a 710-base pair fragment of the mitochondrial cytochrome c oxidase subunit I (COI) gene using specific primers [21]. For polymerase chain reaction (PCR), 5 µL of genomic DNA was amplified using the AccuPower PCR PreMix (Bioneer, Daejeon, Republic of Korea) with the primers listed in S1 Table. The cycling conditions were as follows: initial denaturation at 95°C for 5 min, followed by 35 cycles of denaturation at 95°C for 1 min, annealing at 40°C for 1 min, and extension at 72°C for 30 s, with a final extension at 72°C for 10 min. The resulting amplicons were separated using 1.5% agarose gel electrophoresis and stained with the SYBR® Safe nucleic acid stain (Invitrogen Life Technologies, MA, USA). Amplicons exhibiting the expected DNA molecular weight were gel-purified using the QIAquick Gel Extraction Kit (Qiagen, Hilden, Germany) and sequenced on the Sanger platform. Tick species identification was confirmed by comparing the obtained sequences with reference sequences from GenBank (NCBI, Bethesda, MD, USA).

### Amplification of the 18S rRNA gene and sequencing

Eukaryotic microbiota in the tick samples was detected by amplifying nucleic acid using barcode-tagged primers targeting the V9 region of the 18S rRNA gene, as described by Kim et al. (2022) [22]. The resulting amplicons were pooled and sequenced on an Illumina MiSeq system using the MiSeq Reagent V3 kit (San Diego, CA, USA) following the manufacturer’s instructions. The primer sequences are listed in S1 Table.

### Bioinformatics and statistical analysis

Bioinformatics analysis was conducted using the standard DADA2 denoising pipeline [23] in Quantitative Insights Into Microbial Ecology (QIIME) 2 software, Version 2024.2 [24], for tasks such as demultiplexing, forward and reverse paired-end read merging, quality filtering, and chimeric sequence removal to generate amplicon sequence variant (ASV) feature tables. To classify the taxonomic identities of eukaryotic ASVs, we built a database of fungi and parasites by retrieving relevant sequences from the NCBI nucleotide database (https://www.ncbi.nlm.nih.gov/nuccore/) [25] through an advanced search for “18S rRNA” [26]. Sequences from chordates, plants, and arthropods were excluded to focus on the eukaryotic microbiota and potential pathogens of the ticks. Taxonomic reads were classified using the classify-consensus-blast plugin in QIIME 2.

Alpha diversity was assessed using two metrics—richness (the number of ASVs per sample) and the Shannon diversity index. Differences in the number of observed species and the Shannon index between groups were analyzed using the Wilcoxon rank-sum test. For beta diversity, principal coordinate analysis (PCoA) based on Bray–Curtis distances and permutational multivariate analysis of variance were performed. The Yates-corrected chi-square test was performed in R Studio (Version 2022) to analyze the difference in prevalence between the groups [27]. A *p*-value of <0.05 was considered statistically significant. The map was created using the “ggplot2” library in R [28]. The shapefile was obtained from the Humanitarian Data Exchange (HDX) “Kyrgyzstan - Subnational Administrative Boundaries” dataset, which is publicly available (https://data.humdata.org/dataset/cod-ab-kgz) [29]. Taxa sequences with fewer than five read counts were excluded from the analysis.

### Ethics approval

This study was conducted in accordance with the guidelines set by the Institutional Animal Care & Use Committee (IACUC) of South Korea, as specified by the Joint Authority of the Food and Drug Administration and the Ministry of Agriculture, Food and Rural Affairs. Ethical approval was not required, as the study involved only the collection and analysis of tick samples and did not include direct contact with animals or experimental treatments.

## Results

A total of 472 tick samples were collected during the entire entomological survey. Sanger sequencing identified the ticks as belonging to five genera—*Alveonasus*, *Dermacentor*, *Haemaphysalis*, *Hyalomma,* and *Rhipicephalus*—with 11 tick species identified overall. Among these, *Dermacentor* spp. was the most dominant (28.8%, n = 136) (Table 1). A higher number of ticks were found feeding on cattle (68.9%, n = 325) compared to sheep. Approximately 92% of the collected ticks were in the adult life stage (n = 434), with a slightly higher proportion of males than females (50.5%, n = 219 vs. 49.5%, n = 215, respectively).

**Table 1:** Metadata summary of tick species distribution by host, sex, life stage, and host number in the life cycle.

Species	Host			Life stage			Sex			Host number in the life cycle	Number of samples (%)
	n (%)		*p*-value	n (%)		*p*-value	n (%)		*p*-value		
	Cattle	Sheep		Adult	Nymph		Male	Female			
***Rhipicephalus annulatus***	8 (100)	0 (0.0)	0.005**	2 (25.0)	6 (75.0)	0.157	2 (100)	0 (0.0)	0.157	1 (One-host)	8 (1.6)
***Rhipicephalus turanicus***	24 (44.4)	30 (55.6)	0.414	42 (77.8)	12 (22.2)	0.001***	14 (33.3)	28 (66.7)	0.030*	2 (Two-host)	54 (11.4)
***Rhipicephalus sanguineus***	0 (0.0)	1 (100)	0.317	0 (0.0)	1 (100)	0.317	0 (0.0)	0 (0.0)	–	3 (Three-host)	1 (0.2)
***Dermacentor* spp.**	113 (83.1)	23 (16.9)	0.001***	134 (98.5)	2 (1.5)	0.001***	65 (48.5)	69 (51.5)	0.730	3 (Three-host)	136 (28.8)
***Hyalomma scupense***	64 (100)	0 (0.0)	0.001***	64 (100)	0 (0.0)	0.001***	39 (60.9)	25 (39.1)	0.080	2 (Two-host)	64 (13.6)
***Hyalomma rufipes***	1 (25.0)	3 (75.0)	0.317	4 (100)	0 (0.0)	0.046*	2 (50.0)	2 (50.0)	1	2 (Two-host)	4 (0.8)
***Hyalomma marginatum***	39 (53.4)	34.5 (46.6)	0.558	73 (100)	0 (0.0)	0.001***	44 (60.3)	29 (39.7)	0.080	2 (Two-host)	73 (15.5)
***Hyalomma anatolicum***	15 (100)	0 (0.0)	0.001***	15 (100)	0 (0.0)	0.001***	10 (66.7)	5 (33.3)	0.197	3 (Three-host)	15 (3.2)
***Hyalomma asiaticum***	8 (72.7)	3 (27.3)	0.132	11 (100)	0 (0.0)	0.001***	7 (63.6)	4 (36.4)	0.366	3 (Three-host)	11 (2.3)
***Haemaphysalis punctata***	34 (55.7)	27 (44.3)	0.370	60 (98.4)	1 (1.6)	0.001***	15 (25.0)	45 (75.0)	0.001***	3 (Three-host)	61 (12.9)
***Alveonasus lahorensis***	19 (42.2)	26 (57.8)	0.297	29 (64.4)	16 (35.6)	0.053	21 (72.4)	8 (27.6)	0.016*	2 (Two-host)	45 (9.5)

Notes: Yates-corrected chi-square test was used. *p < 0.05, **p < 0.01, ***p < 0.001. Only the adult tick life stages were considered for the sex distribution column

**Fig 2:**
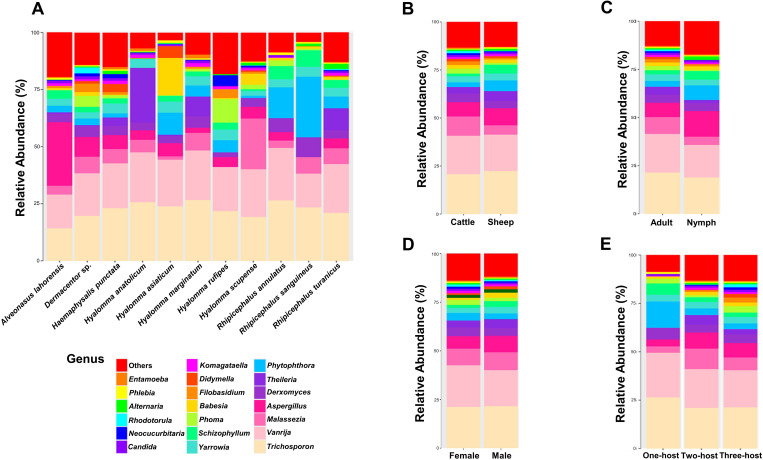
Average relative abundance of eukaryotic microbial taxa. Bar plots show the average relative abundance of eukaryotic microbial taxa based on tick characteristics: (A) species, (B) hosts, (C) life stage, (D) sex, and (E) host number in the life cycle.

Sequencing of the eukaryotic 18S rRNA V9 region yielded 2,081,305 raw amplicon reads, with an average depth of 4,410 reads per sample, identifying a total of 265 microeukaryotic entities (S2 Table). The method detected known protozoan pathogens such as *Babesia* and *Theileria*, as well as environmental fungal species like *Mortierella* [30]. Bar graphs illustrating relative abundance revealed the top 20 most abundant taxa, with distinct relative patterns across tick species, host, life stage, sex, and host number in the life cycle (Figs 2A–E). Among these, three genera were protozoan (*Babesia*, *Theileria*, *Entameba*), while the remaining 17 taxa were fungi. Alpha diversity analysis revealed significantly higher diversity in ticks collected from sheep, female ticks (based on the Shannon index), and nymph-stage ticks (S1 Fig). Beta diversity analysis using the Bray–Curtis distance index showed that the composition of the eukaryotic microbiota was significantly influenced by tick host, life stage, sex, and host number in the life cycle (S2 Fig).

We observed the detection rate of two potentially pathogenic eukaryotes, *Babesia* and *Theileria*, across the surveyed areas. Overall, 13.3% (63/472) of the ticks tested positive for *Babesia* and 12.7% (60/472) for *Theileria* 18S rRNA genomic sequences (Table 2). All identified tick species, except *Hyalomma anatolicum,* tested positive for *Babesia,* with detection rates ranging from 6.6% to 100%. Conversely, *Theileria* was detected in seven tick species, with detection rates varying from 2.2% to 46.7%. Moreover, the detection rates of *Babesia* and *Theileria* were examined based on tick host, life stage, sex, and host number in the life cycle, as shown in Table 3. The results revealed that the detection rate of *Babesia* was significantly higher in sheep (23.8%) than in cattle (8.6%). Furthermore, *Babesia* was more commonly detected in the nymph-stage ticks (42.1%) than in the adult tick life stage (10.8%). However, the detection rate of *Theileria* did not show significant differences based on tick-related variables.

**Table 2:** Detection rates of potential pathogens in different tick species.

Species	Tested samples (n)	*Babesia*			*Theileria*		
		Positive (n)	Detection rate (%)	*p*-value	Positive (n)	Detection rate (%)	*p*-value
***Rhipicephalus annulatus***	8	3	37.5	0.078	0	0.0	0.604
***Rhipicephalus turanicus***	54	10	18.5	0.001***	16	29.6	0.002**
***Rhipicephalus sanguineus***	1	1	100	0.133	0	0.0	n. a.
***Dermacentor* spp.**	136	14	10.3	0.001***	3	2.2	0.001***
***Hyalomma scupense***	64	5	7.8	0.001***	0	0.0	n. a.
***Hyalomma rufipes***	4	1	25.0	0.437	0	0.0	n. a.
***Hyalomma marginatum***	73	8	11.0	0.001***	14	19.2	0.001***
***Hyalomma anatolicum***	15	0	0.0	n. a.	7	46.7	0.796
***Hyalomma asiaticum***	11	5	45.5	0.763	1	9.1	0.007**
***Haemaphysalis punctata***	61	4	6.6	0.001***	14	23.0	0.001***
***Alveonasus lahorensis***	45	12	26.7	0.002**	5	11.1	0.001***
**Total**	472	63	13.3		60	12.7	

Note: The Yates-corrected chi-square test was used. *p < 0.05, **p < 0.01, ***p < 0.001.

**Table 3:** Detection rates of potential pathogens based on tick host, life stage, sex, and host number in the life cycle.

Variable	Category	Tested samples (n)	*Babesia*			*Theileria*		
			Positive (n)	Detection rate (%)	*p-*value	n	Detection rate (%)	*p*-value
							Positive	
**Host**	Cattle	325	28	8.6	0.001***	35	10.8	0.083
	Sheep	147	35	23.8		25	17.0	
**Life stage**	Adult	434	47	10.8	0.001***	56	12.9	0.867
	Nymph	38	16	42.1		4	10.5	
**Sex**	Male	219	23	10.5	0.947	23	10.5	0.173
	Female	215	24	11.2		33	15.3	
**Host number in the life cycle**	One-host	8	3	37.5	0.051	0	0.0	0.3
	Two-host	240	36	15.0		35	14.6	
	Three-host	224	24	10.7		25	11.2	

Note: Yates-corrected chi-square test test was used. *p < 0.05, **p < 0.01, ***p < 0.001.

**Fig 3:**
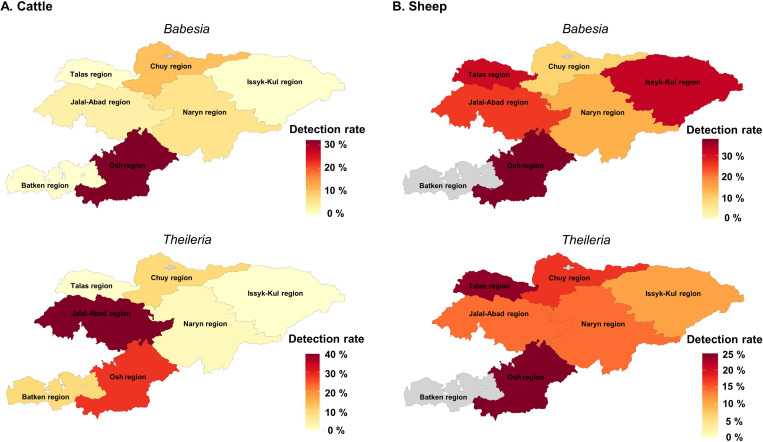
Detection rates of *Babesia* and *Theileria* in ticks across Kyrgyzstan. The maps show the detection rates of *Babesia* and *Theileria* in ticks, with a color gradient ranging from high (red) to low (yellow) detection rates. **(A)** *Babesia* (top) and *Theileria* (bottom) in cattle ticks. **(B)** *Babesia* (top) and *Theileria* (bottom) in sheep ticks. The base map was adapted for illustrative purposes from administrative boundary data provided by the United Nations Office for the Coordination of Humanitarian Affairs (OCHA).

The counts of potentially pathogenic *Babesia* and *Theileria* recovered from cattle and sheep tick surveys, respectively, and categorized based on geographical regions, are summarized in Tables 4 and 5. Overall, both potential pathogens showed high detection rates in the Osh region, particularly in sheep samples (Fig 3). Ticks collected from cattle in the Jalal-Abad region showed the highest *Theileria* detection rate (40.0%), while the Osh region had the highest *Babesia* detection rate (32.0%) (Table 4). Among ticks collected from sheep in the Osh region showed the highest *Theileria* detection rate (25.0%), whereas the lowest *Babesia* detection rate (10.0%) was observed in the Chuy region (Table 5).

**Table 4:** Detection rates of potential pathogens in ticks collected from cattle by geographical region.

Region	Tested samples (n)	*Babesia*		*Theileria*	
**Positive (n)**	**Detection rate (%)**	**Positive (n)**	**Detection rate (%)**
**1. Batken**	21	0	0.0	2	9.5
**2. Osh**	25	8	32.0	7	28.0
**3. Jalal-Abad**	30	1	3.3	12	40.0
**4. Talas**	10	0	0.0	0	0.0
**5. Chuy**	123	13	10.6	12	9.8
**6. Naryn**	103	6	5.8	2	1.9
**7. Issyk-Kul**	13	0	0.0	0	0.0

**Table 5:** Detection rates of potential pathogens in ticks collected from sheep by geographical region.

Region	Tested samples (n)	*Babesia*		*Theileria*	
		Positive (n)	Detection rate (%)	Positive (n)	Detection rate (%)
**1. Batken**	0	–	–	–	–
**2. Osh**	8	3	37.5	2	25.0
**3. Jalal-Abad**	35	9	25.7	5	14.3
**4. Talas**	29	9	31.0	7	24.1
**5. Chuy**	40	4	10.0	7	17.5
**6. Naryn**	7	1	14.3	1	14.3
**7. Issuk-Kul**	28	9	32.1	3	10.7

## Discussion

Ticks are not only blood-sucking ectoparasites but also key vectors of TBDs, posing significant risks to both public and animal health. Notably, ticks can be introduced into new regions through the movement of infested livestock and wildlife, facilitating the spread of TBDs across borders [31]. Therefore, understanding tick populations and infection patterns in Kyrgyzstan also has implications for neighboring countries.

While previous studies have documented the distribution of ticks and some tick-borne diseases in Kyrgyzstan [12,32–34], research remains limited on the relationship between tick-related factors (e.g., host, life stage, sex, and host number in the life cycle) and the presence of eukaryotic pathogens. This study is the first nationwide investigation to analyze these relationships using eukaryotic NGS metabarcoding in Kyrgyzstan.

In this study, 472 ticks representing 11 species from five genera were collected from cattle and sheep across grazing fields in seven regions of Kyrgyzstan—*Dermacentor* spp., *Hyalomma marginatum*, *Hyalomma scupense*, *Haemaphysalis punctata*, *Rhipicephalus turanicus*, *Alveonasus lahorensis*, *Hyalomma anatolicum*, *Hyalomma asiaticum*, *Rhipicephalus annulatus*, *Hyalomma rufipes*, and *Rhipicephalus sanguineus* (Table 1). These findings align with those of Aknazarov et al. (2023), Fedorova (2005), and Kim et al. (2024), who also reported similar distributions of these tick species across Kyrgyzstan [32,33,35]. Tick abundance, categorized by genus and species, varied significantly across the seven biogeographic zones and was defined by distinct climatic and ecological characteristics [36,37]. As ectoparasites, ticks exhibit remarkable adaptability to different animal hosts and often show specific host preferences [32,37,38].

Taxonomic analysis of the V9 region of the 18S rRNA gene sequences identified 265 taxa hosted by tick vectors. Among these, two protozoa, *Babesia* and *Theileria*, known for their pathogenic potential, were the most prevalent [39–41]. Previous studies have linked variations in the relative abundance of microbiota constituents to factors such as life stage [42], sex [43], tick species [44,45], and tick life cycle phases [46]. In this study, *Babesia* showed a higher detection rate in ticks collected from sheep, indicating differences in *Babesia* prevalence across animal hosts within the ecological context of Kyrgyzstan.

In our study, the prevalence rate of *Babesia* was significantly higher in nymphal stage. Larval ticks often acquire *Babesia* from small mammal reservoir hosts, such as rodents, during their initial blood meal [47]. The infection is then efficiently passed on to the nymphal stage through transstadial transmission, a critical aspect of *Babesia* epidemiology [48]. Consequently, nymphs are the first stage potentially carrying pathogens acquired from key *Babesia* reservoir hosts during their larval phase. Both nymph and adult ticks can transmit the causative agents of babesiosis [49], though, in some *Babesia* species, nymphs are more efficient at transmission than adults [50]. Furthermore, nymphal salivary glands are often more intensely parasitized than those in adults [51,52]. These findings align with the results of this study, and the differences in detection rates between *Theileria* and *Babesia* may reflect these dynamics. While the effects of certain variables were observed, future studies should control for additional factors to isolate each effect, as tick microbiome composition is known to vary accordingly. Further research is needed to explore the underlying reasons for these variations across diverse ecological contexts.

In addition to identifying the drivers of pathogen distribution, it is essential to evaluate whether the detection rates for *Babesia* and *Theileria* observed in this nationwide survey reflect stable endemicity in Kyrgyzstan or represent a transient pattern specific to the March–July 2022 sampling period. As this is the first comprehensive national study using NGS metabarcoding to investigate eukaryotic tick-borne pathogens in the country, the lack of historical longitudinal data precludes definitive conclusions about the long-term prevalence of these pathogens. Nevertheless, despite the limitations of a single survey period, our findings provide a critical initial epidemiological baseline for future surveillance and comparative studies. It is well-documented that climate change is a significant driver altering tick-borne disease risk globally [53–55], with rising temperatures accelerating tick development, lengthening activity seasons, and enabling range expansion to higher latitudes and altitudes [53,55]. Such northward and upward shifts have been recorded for species like *Ixodes scapularis* in Canada and *Ixodes ricinus* in Europe [54,55]. Furthermore, climate-driven shifts in tick phenology can alter pathogen transmission dynamics by affecting the temporal overlap of different life stages and their hosts [54,55]. Therefore, monitoring the tick and pathogen populations identified in our study over time is essential for predicting future disease hotspots and adapting public health and veterinary control strategies to a changing regional environment.

In this study, the significantly higher detection rate of *Babesia* in ticks collected from sheep (23.8%) compared to those from cattle (8.6%) (Table 3) prompts an examination of whether this is a common pattern or more specific to the ecological context of Kyrgyzstan. Data from other countries indicates considerable variability in *Babesia* prevalence related to sheep. For instance, a study from Dehgolan, Iran, presents a scenario where high *Babesia* circulation in sheep appears to correspond with significant tick infection rates [56]. That study reported a notable overall *Babesia* prevalence of 14.15% in sheep hosts and very high *Babesia* spp. infection in tick genera known to infest sheep, such as *Rhipicephalus* (e.g., *R. bursa* 63.95% positive) and *Haemaphysalis punctata* (36.11% positive) [56]. While a direct comparison of *Babesia* prevalence in ticks collected specifically from sheep versus specifically from cattle was not made in that study, the high prevalence in sheep hosts and in sheep-associated tick species in their study area suggests that the high rate (23.8%) observed in ticks from sheep in Kyrgyzstan could occur in regions with substantial sheep babesiosis.

In contrast, other studies indicate much lower *Babesia* pressure in sheep. For example, research in other Iranian provinces found no *Babesia* DNA in sheep blood samples (0/95), while cattle in the same study showed 7.10% positivity [57]. Similarly, a study in Mosul, Iraq, reported a very low *Babesia* prevalence of 0.01% in sheep hosts [58]. These contrasting findings suggest that high *Babesia* prevalence in ticks from sheep is not universal and likely depends on local factors. Therefore, the higher *Babesia* detection in ticks from sheep in our study may reflect specific local epidemiological factors in Kyrgyzstan, the particular competency of local tick vectors, and distinct ecological interactions that favor transmission in sheep.

The prevalence of *Babesia* (13.3%) and *Theileria* (12.7%) in ticks from this Kyrgyzstan study serves as a key regional reference, highlighting significant pathogen presence. Comparative data from neighboring countries reveals a complex epidemiological landscape. In northwestern China, one study investigating 1,084 adult ticks from 11 border counties or cities reported an overall prevalence of approximately 1.01% for *Babesia* (11 positive samples) and 0.65% for *Theileria* (7 positive samples) [59]. The rates observed in Chinese ticks are notably lower than the *Babesia* and *Theileria* prevalence reported in this study. However, other studies from the same border regions of northwestern China focusing on sheep show a different picture. For example, a study of 323 Chinese sheep found that infection rates for *Babesia motasi*-like (18.6%) and *Theileria uilenbergi* (15.8%) were comparable to the prevalence in Kyrgyz ticks, highlighting the need for caution when comparing host and vector data [60]. From Kazakhstan, one molecular survey of ticks reported an overall *Theileria* spp. prevalence of 4.6% in *Dermacentor marginatus*, a rate notably lower than the 12.7% we detected for *Theileria* spp. reported in this study [61]. Furthermore, a different molecular survey in Kazakh cattle found an overall *Babesia* spp. prevalence of 24.7%, a considerably higher rate than the 13.3% prevalence detected in ticks in this study [62].

Collectively, comparing our findings with regional data underscores the widespread distribution of *Babesia* and *Theileria* across Central Asia, though direct comparisons of prevalence rates must be approached with caution due to differences in sample types (i.e., ticks from our study versus animal blood or serum in others), vertebrate host species (e.g., cattle or sheep), diagnostic methodologies (NGS metabarcoding versus targeted PCR or serology), sampling strategies, and specific ecological niches. Despite these caveats, the comparisons illustrate a variable epidemiological picture: in northwestern China, while piroplasm prevalence in ticks [59] was notably lower than in ticks collected from Kyrgyzstan, studies in Chinese sheep [60] indicated higher prevalence for certain species of piroplasms. Similarly, in Kazakhstan, the prevalence of *Theileria* in certain species of ticks was reported to be lower (e.g., 4.6% in *Dermacentor marginatus* ticks) than in ticks from Kyrgyzstan [61], whereas the molecular prevalence of *Babesia* in Kazakh cattle was considerably higher (24.7%) [62]. The consistent detection of these pathogens across borders, even with differing prevalence rates, points to shared risk factors, such as similar tick vector populations and livestock, which are pertinent for transboundary disease surveillance and control efforts. The insights gained from such regional comparisons emphasize the need for harmonized surveillance approaches and collaborative research initiatives among neighboring countries to better understand the complex epidemiology of these economically important tick-borne parasitic diseases and to develop effective regional control strategies.

This study has several limitations, including the resolution of the Illumina MiSeq system, which hindered species-level identification, and the absence of detailed host data across diverse ecological contexts and climates. Consequently, assessing the specific zoonotic risk of *Babesia* and *Theileria* species to humans or their impact on different livestock hosts is limited by the current resolution. Moreover, while *Babesia* prevalence was higher in nymphs than adults, limited number of nymphal samples for most tick species prevented species-specific analysis. Future studies with more extensive nymphal sampling across various tick species would be valuable to investigate species-specific dynamics. Furthermore, while the ticks were washed after the collection, it is plausible that some detected environmental DNA originated from the external surface of the ticks rather than from internal eukaryotic microorganisms, as some residual DNA from environmental organisms, including fungi or protist, may persist despite washing. Future research should employ advanced sequencing technologies alongside comprehensive environmental and behavioral analyses to deepen our understanding of these complex interactions. Additionally, further studies are needed on the eukaryotic microbiota of both the internal and external surfaces of ticks to identify fungi and *Entameba* within them, which would help to clarify the origin of such microorganisms and distinguish between surface contaminants and true internal constituents.

## Conclusions

In conclusion, this study is the first comprehensive nationwide investigation of eukaryotic potential pathogens in ticks in Kyrgyzstan using metabarcoding. Our findings provide crucial insights into tick epidemiology and its relationships with host characteristics in Kyrgyzstan. By assessing prevalence at the genus level across multiple tick species and livestock hosts nationwide, this study offers a broader view of the overall ecological dynamics and distribution of *Babesia* and *Theileria* than captured by previous, more targeted species-specific surveys. Given the direct impact of TBDs on livestock health, productivity, and welfare, our findings have significant implications for the agricultural sector. Understanding the distribution and prevalence of these pathogens is essential for improving livestock management and mitigating economic losses in Kyrgyzstan. This research provides the basis for strategic public health initiatives and the development of effective control measures for tick pests and TBDs in the region.

## Supporting information

S1 Fig Visualization of alpha diversity in ticks based on their characteristics. **(A)** Box plot comparing the observed characteristics of eukaryotic microbial diversity in ticks from cattle and sheep. **(B)** Box plot comparing the Shannon index of eukaryotic microbial diversity in ticks from cattle and sheep. **(C)** Box plot comparing the observed characteristics of eukaryotic microbial diversity between male and female ticks. **(D)** Box plot comparing the Shannon index of eukaryotic microbial diversity between male and female ticks. **(E)** Box plot comparing the observed characteristics of eukaryotic microbial diversity between adult and nymph ticks. **(F)** Box plot comparing the Shannon index of eukaryotic microbial diversity between adult and nymph ticks. **(G)** Box plot comparing the observed characteristics of eukaryotic microbial diversity in ticks based on host number in the life cycle. **(H)** Box plot comparing the Shannon index of eukaryotic microbial diversity in ticks based on host number in the life cycle. The alpha diversity indices (Shannon index and observed characteristics) were analyzed using the Wilcoxon rank-sum test.
(TIFF)

S2 Fig Principal coordinates analysis (PCoA) plot showing beta diversity (Bray–Curtis distance) in ticks based on their characteristics. **(A)** PCoA plot depicting eukaryotic microbial diversity in ticks from cattle and sheep. **(B)** PCoA plot depicting eukaryotic microbial diversity in adult and nymph ticks. **(C)** PCoA plot depicting eukaryotic microbial diversity in male and female ticks. **(D)**PCoA plot depicting eukaryotic microbial diversity in ticks based on host number in the life cycle. The beta diversity index (Bray–Curtis distance) was analyzed using permutational multivariate analysis of variance (PERMANOVA).
(TIFF)

S1 Table List of primers used in this study. (DOCX)

S2 Table Taxa of eukaryotic microbiota and metadata of ticks. (XLSX)
