# Supplementary figures and images for: Nationwide investigation of eukaryotic pathogens in ticks from cattle and sheep in Kyrgyzstan using metabarcoding
Source: PLoS One. 2025 Aug 5;20(8):e0327953. doi: 10.1371/journal.pone.0327953 (PMC12324094; doi:10.1371/journal.pone.0327953)

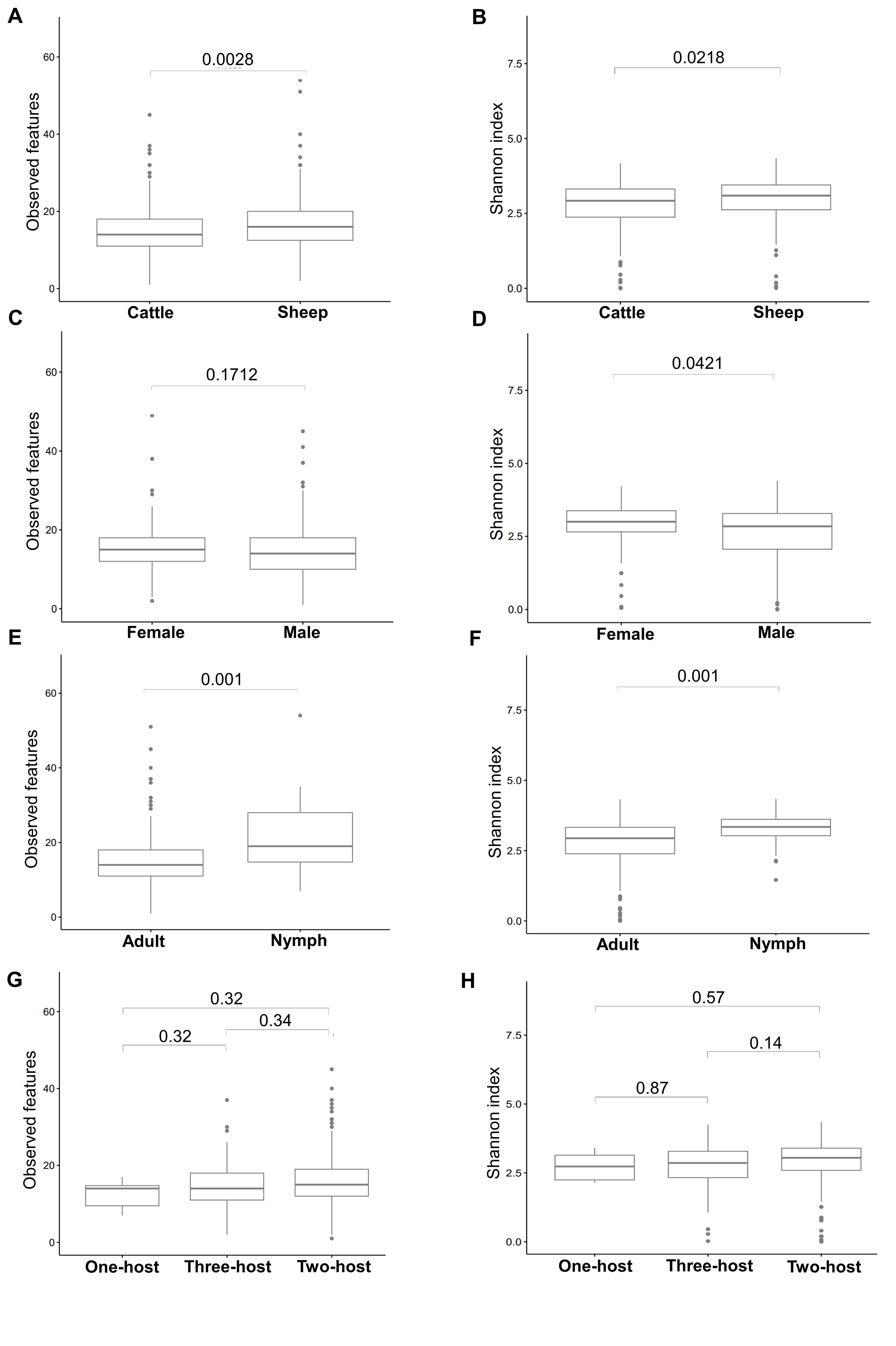

Supplement: S1 Fig — (A) Box plot comparing the observed characteristics of eukaryotic microbial diversity in ticks from cattle and sheep. (B) Box plot comparing the Shannon index of eukaryotic microbial diversity in ticks from cattle and sheep. (C) Box plot comparing the observed characteristics of eukaryotic microbial diversity between male and female ticks. (D) Box plot comparing the Shannon index of eukaryotic microbial diversity between male and female ticks. (E) Box plot comparing the observed characteristics of eukaryotic microbial diversity between adult and nymph ticks. (F) Box plot comparing the Shannon index of eukaryotic microbial diversity between adult and nymph ticks. (G) Box plot comparing the observed characteristics of eukaryotic microbial diversity in ticks based on host number in the life cycle. (H) Box plot comparing the Shannon index of eukaryotic microbial diversity in ticks based on host number in the life cycle. The alpha diversity indices (Shannon index and observed characteristics) were analyzed using the Wilcoxon rank-sum test. (TIFF) [file pone.0327953.s001.tiff]

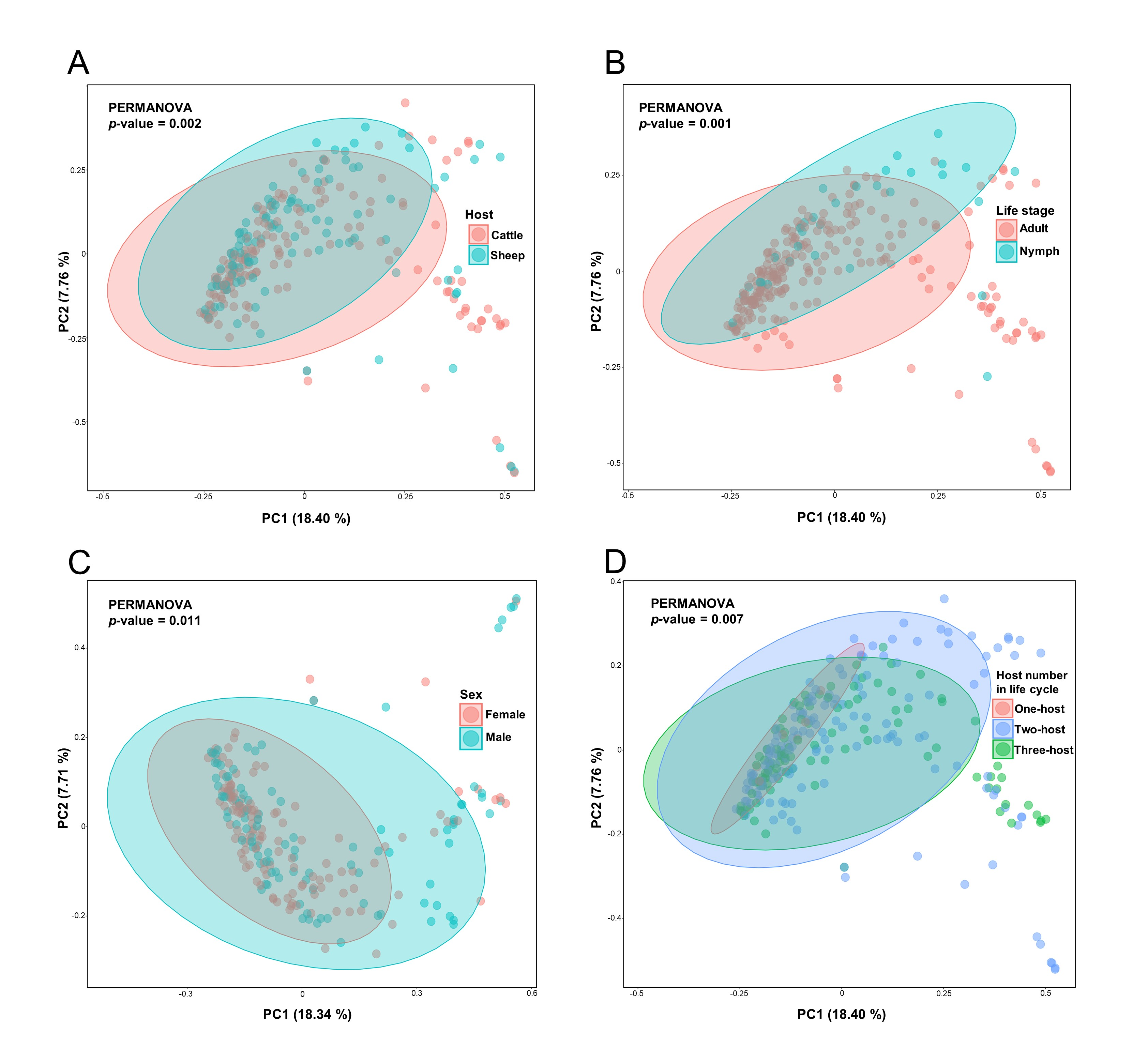

Supplement: S2 Fig — (A) PCoA plot depicting eukaryotic microbial diversity in ticks from cattle and sheep. (B) PCoA plot depicting eukaryotic microbial diversity in adult and nymph ticks. (C) PCoA plot depicting eukaryotic microbial diversity in male and female ticks. (D)PCoA plot depicting eukaryotic microbial diversity in ticks based on host number in the life cycle. The beta diversity index (Bray–Curtis distance) was analyzed using permutational multivariate analysis of variance (PERMANOVA). (TIFF) [file pone.0327953.s002.tiff]
