# Supplementary material for: Nationwide investigation of eukaryotic pathogens in ticks from cattle and sheep in Kyrgyzstan using metabarcoding
Source: PLoS One. 2025 Aug 5;20(8):e0327953. doi: 10.1371/journal.pone.0327953 (PMC12324094; doi:10.1371/journal.pone.0327953)
Supplement: S1 Table — (DOCX) [file pone.0327953.s003.docx]

**Supplementary Table 1.** List of primers used in this study

| **Gene** | **Primer** | **Sequence (5**′**→ 3**′**)** |
| --- | --- | --- |
| COI | LCO1490 | GGTCAACAAATCATAAAGATATTGG |
|  | HCO2198 | TAAACTTCAGGGTGACCAAAAAATCA |
| 18S rRNA gene V9 | 1391f | TCGTCGGCAGCGTCAGATGTGTATAAGAGACAG GTACACACCGCCCGTC |
|  | EukBr | GTCTCGTGGGCTCGGAGATGTGTATAAGAGACAGTGATC CTTCTGCAGGTTCACCTAC |
